# Supplementary material for: Different Dose of Sucrose Consumption Divergently Influences Gut Microbiota and PPAR-γ/MAPK/NF-κB Pathway in DSS-Induced Colitis Mice
Source: Nutrients. 2022 Jul 4;14(13):2765. doi: 10.3390/nu14132765 (PMC9268685; doi:10.3390/nu14132765)
Supplement: Supplementary file 1 [file nutrients-14-02765-s001.zip › nutrients-1742415-supplementary.pdf]

Supporting Information

Divergently Regulation of Different Dose Sucrose Consumption on Gut Microbiota and PPAR-γ/MAPK/NF-κB Pathway in DSS-Induced Colitis Mice

List of content

Supplementary Table S1. Disease Activity Index Score

Supplementary Table S2. Sequence of the primers used in this study [sequence 5'-3']

Supplementary Table S1. Disease Activity Index Score

Disease Activity Index Score			
**score**	**Rectal bleeding**	**Stool consistency**	**Weight loss（%）**
**0**	none	normal	0
**1**	occult blood negative	soft but formed	1-5
**2**	occult blood positive slightly	loose	6-10
**3**	occult blood positive	mild diarrhea	11-15
**4**	gross bleeding	severe diarrhea	> 15

The final macroscopic score for each animal is the average of these three separate scores.

Supplementary Table S2. Sequence of the primers used in this study [sequence 5'-3']

Gene	Forward	Reverse
β-actin	CTACCTCATGAAGATCCTGACC	CACAGCTTCTCTTTGATGTCAC
IL-6	CTCCCAACAGACCTGTCTATAC	CCATTGCACAACTCTTTTCTCA
TNF-α	ATGTCTCAGCCTCTTCTCATTC	GCTTGTCACTCGAATTTTGAGA
IL-1β	AAGGGCTGCTTCCAAACCTTTGAC	TGCCTGAAGCTCTTGTTGATGTGC
ZO-1	CTGGTGAAGTCTCGGAAAAATG	CTGGTGAAGTCTCGGAAAAATG
Occludin	ACG GAC CCT GAC CAC TAT GA	TCA GCA GCA GCC ATG TAC TC
Claudin-1	AGATACAGTGCAAAGTCTTCGA	CAGGATGCCAATTACCATCAAG
PPAR-γ	CCAAGAATACCAAAGTGCGATC	TCACAAGCATGAACTCCATAGT
